# Topological data analysis reveals core heteroblastic and ontogenetic programs embedded in leaves of grapevine (Vitaceae) and maracuyá (Passifloraceae)

**DOI:** 10.1371/journal.pcbi.1011845

**Published:** 2024-02-05

**Authors:** Sarah Percival, Joyce G. Onyenedum, Daniel H. Chitwood, Aman Y. Husbands

**Affiliations:** 1 Department of Computational Mathematics, Science & Engineering, Michigan State University, East Lansing, Michigan, United States of America; 2 Department of Environmental Studies, New York University, New York, New York, United States of America; 3 Department of Horticulture, Michigan State University, Michigan State University, East Lansing, Michigan, United States of America; 4 Department of Biology, University of Pennsylvania, Philadelphia, Pennsylvania, United States of America; 5 Epigenetics Institute, University of Pennsylvania, Philadelphia, Pennsylvania, United States of America; Universitat zu Koln, GERMANY

## Abstract

Leaves are often described in language that evokes a single shape. However, embedded in that descriptor is a multitude of latent shapes arising from evolutionary, developmental, environmental, and other effects. These confounded effects manifest at distinct developmental time points and evolve at different tempos. Here, revisiting datasets comprised of thousands of leaves of vining grapevine (Vitaceae) and maracuyá (Passifloraceae) species, we apply a technique from the mathematical field of topological data analysis to comparatively visualize the structure of heteroblastic and ontogenetic effects on leaf shape in each group. Consistent with a morphologically closer relationship, members of the grapevine dataset possess strong core heteroblasty and ontogenetic programs with little deviation between species. Remarkably, we found that most members of the maracuyá family also share core heteroblasty and ontogenetic programs despite dramatic species-to-species leaf shape differences. This conservation was not initially detected using traditional analyses such as principal component analysis or linear discriminant analysis. We also identify two morphotypes of maracuyá that deviate from the core structure, suggesting the evolution of new developmental properties in this phylogenetically distinct sub-group. Our findings illustrate how topological data analysis can be used to disentangle previously confounded developmental and evolutionary effects to visualize latent shapes and hidden relationships, even ones embedded in complex, high-dimensional datasets.

## Author summary

Questions in biology are increasingly driven by large datasets comprised of disparate types of data obtained through ecological, morphological, and molecular measurements. A key challenge in the field is thus to make biologically meaningful sense of this enormous amount of data. Methods in topological data analysis offer a flexible and powerful solution to this challenge. To illustrate this, we interrogated datasets of grapevine and maracuyá (passion-flower) leaves using the Mapper algorithm, a method of topological data analysis that presents hidden relationships in an easily visualizable graph or network. Our analyses identified core, deeply conserved developmental signatures in all species of grapevine and most species of maracuyá that were not detected using traditional analyses. We also found two interesting exceptions to this trend in the maracuyá family. These species showed a ‘reverse hourglass’ effect, suggesting their developmental programs have been modified but only at nodes near the middle of the leaf series. As these exceptions cluster phylogenetically, we propose an independently evolving heteroblasty program may be at play in this subclade. Our analyses illustrate the power of topological data analysis to isolate signatures normally hidden within high-dimensional datasets, and to identify biologically relevant exceptions to those specific signatures.

## Introduction

Leaf shape is dynamic. Rather than viewing the ways it changes in response to evolutionary, developmental, and environmental forces as facets of a single form, we partition these effects separately from each other. This was not always the case, and in early philosophical conceptualizations of the plant form, evolutionary, developmental, and environmental responses flowed seamlessly with each other, focusing on the organismal form [1]. Before Darwin, the idea of gradual change as the foundation of evolutionary thinking was elaborated by Goethe, focusing on the metameric, serial homology between leaves and other plant organs [1]. In describing changes to mature leaf shape across sequential nodes, as well as more dramatic metamorphoses of lateral organs, Goethe declared that the ideal leaf is mutable and its only constant is change itself: “*daß in demjenigen Organ der Pflanze*, *welches wir als Blatt gewöhnlich anzusprechen pflegen*, *der wahre Proteus verborgen liege*” (“that in the organ of the plant which we are accustomed to calling the leaf, the true Proteus lies hidden” [2]). Experiments by Hales pricking a grid of points in a young fig leaf and measuring vertical and horizontal displacement as it expanded determined that, not only does leaf shape change across successive nodes, but that the shape of each individual leaf constantly changes during its development: “By observing the difference of the progressive and lateral motions of these points in different leaves, that were of very different lengths in proportion to their breadths.” [3]. The notion that leaves have different ontogenetic programs depending on their node is termed heteroblasty [4]. This concept can be confusing as terms like ‘young’ and ‘old’, or ‘juvenile’ and ‘adult’, are often imprecisely defined. Consider the first leaf produced on a plant. This leaf is born, matures, and dies, in a process termed ontogeny. All leaves undergo ontogenesis; however, the nature of the ontogenetic program differs depending on when the leaf was produced. For instance, the first leaf produced on a plant is considered juvenile regardless of its ontogenetic age, as it was produced when the plant itself was juvenile. Ontogenetic programs are thus partly defined by heteroblasty, and these processes can be easily confounded in actively growing leaves. The environment, too, modulates leaf shape, acting on both heteroblastic and ontogenetic processes. Evolution and the environment thus act on multiple developmental processes, including ontogeny and heteroblasty, to generate inter-species, intra-species, intra-individual, and intra-leaf variation in shape [5]. Discerning the relative contributions of these developmental, environmental, and evolutionary forces in leaf morphogenesis remains a key challenge.

We think of leaf shape geometrically, in terms of the relative size and distance of features to each other [6]. Lobes, serrations, and leaf dimensions are all examples of this geometry. Landmarks–homologous points found on every leaf–allow geometric features to be quantified [7]. For instance, Generalized Procrustes Analysis (GPA) can be used to superimpose leaves on each other [8] using transposition, scaling, and rotation to minimize the distance of landmarks on every leaf to each other. Once superimposed, *x*- and *y*-coordinate values can be modeled and analyzed statistically. Using these methods on grapevine and maracuyá leaves, evolutionary [9,10], developmental [10,11], and environmental effects [12,13,14] can be studied. Ultimately these approaches are statistical and treat each leaf as a separate sample. Population parameters like mean and standard deviation of coordinate values are calculated as summaries and dimension reduction is used to efficiently analyze multivariate data. However, each leaf remains a separate entity from every other, and only the statistical parameters at a population level are modeled.

Just as features within a single leaf have a relative distance to each other, each leaf has a relative distance to other leaves, based on their overall similarity. By computing the correlation distance between each leaf shape, a matrix of all the distances from each leaf to every other leaf can be generated. This distance matrix itself has a visualizable structure: in effect, a “shape of shapes”. Importantly, this structure changes depending on the mathematically defined perspective from which we view it. Topological data analysis is a mathematical field that measures the structure of data by its topology–connected components, loops, voids and other robust features that only change by tearing or detachment (see [15] for a brief overview and [16] for a more thorough treatment). The Mapper algorithm [17] is a method from topological data analysis that visualizes data structures as graphs or networks. The graph structure is primarily determined by a lens function: a real number value assigned to each data point that determines the data structure from a mathematically defined perspective. Mapper has been used to visualize biological data structures from functional- and hypothesis-driven perspectives, including the discovery of cancer-associated genes [18] and discerning developmental transitions in single-cell transcriptomic studies [19].

**Fig 1:**
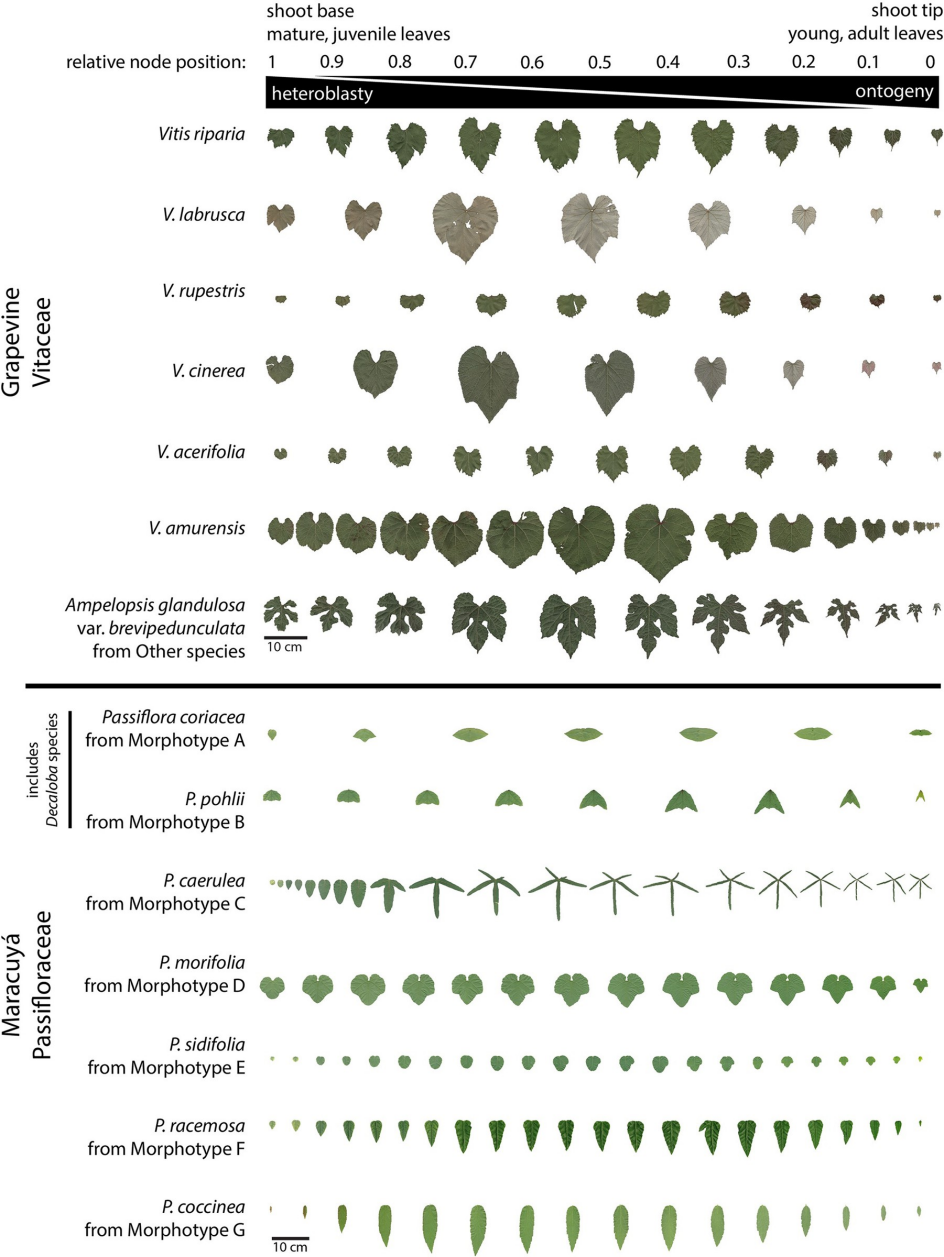
Leaves of grapevine and maracuyá separated by relative node position. Leaves are arranged from shoot base (mature, juvenile) to shoot tip (young, adult). Relative node positions are then assigned for each leaf with 1 being base and 0 being tip. Heteroblasty is evident for all species but is particularly striking in the maracuyá group. Species-to-species differences in leaf morphology is also more dramatic in the maracuyá group. Scale bar = 10cm.

Here, we use topological data analysis to identify developmental and evolutionary relationships hidden within datasets of leaf shape in grapevine (Vitaceae) and maracuyá (Passifloraceae). Both families are noted for the disparate leaf shapes that characterize different species, with maracuyá in particular displaying extreme differences in leaf shape thought to arise from selective pressures of *Heliconius* butterflies laying eggs on its leaves (**Fig 1;** [20]). Using the relative node position of leaves within the shoot as a lens function, we comparatively visualized the developmental progression of leaf shape in each family as a Mapper graph. Our analyses suggest that leaves of grapevine species progress through nearly identical heteroblastic and ontogenetic programs. Surprisingly, heteroblastic and ontogenetic progression in maracuyá species is also strongly conserved despite the strikingly different leaf shapes in this charismatic family. This suggests the acquisition of new morphologies in groups such as maracuyá may result from contributions orthogonal to deeply conserved developmental programs, like heteroblasty and ontogeny, rather than by genetic alterations to the programs themselves. Our analyses also identify two interesting exceptions to this conservation which cluster in subgenus *Decaloba* of maracuyá.

These species appear to have modified their developmental programs, but only at nodes near the middle of the shoot, creating a “reverse hourglass” effect. Taken together, our findings illustrate the power of topological data analysis to isolate conserved developmental relationships hidden within large, high-dimensional datasets, with implications for the development of predictive models of complex phenotypes.

## Materials and methods

### Plant materials

Grapevine data is previously described [9,13,14]. Original leaf scans and landmark data are both publicly available (https://datadryad.org/stash/dataset/doi:10.5061/dryad.zkh189377) [21]. More than 8,400 leaves were collected from up to 11 species and four hybrids representing 208 vines over four different years keeping track of the node, counting from the growing tip of the vine, they were collected from. The species and hybrids are as follows: *Vitis riparia*, *V*. *labrusca*, *V*. *cinerea*, *V*. *rupestris*, *V*. *acerifolia*, *V*. *amurensis*, *V*. *vulpina*, *V*. *aestivalis*, *V*. *palmata*, *V*. *coignetiae*, *Ampelopsis glandulosa* var. *brevipedunculata*, *V*. ×*andersonii*, *V*. ×*champinii*, *V*. ×*doaniana*, *V*. ×*novae-angliae*, and 13 vines with unassigned identity (indicated as *Vitis* spp.). To help simplify visualization, only the six most well-sampled species (*V*. *riparia*, *V*. *acerifolia*, *V*. *labrusca*, *V*. *amurensis*, *V*. *rupestris*, and *V*. *cinerea*) are separately indicated in plots and the remainder are grouped as “Other” (**Fig 1**).

Here we use the Hispanicized name maracuyá, derived from Old Tupí or Guaraní, rather than Passifloraceae, to acknowledge and honor the Indigenous cultures that first described these species [22]. Maracuyá data is previously described [10,23]. Original leaf scans and landmark data are both publicly available (https://datadryad.org/stash/dataset/doi:10.5061/dryad.zkh189377) [21]. More than 3,300 leaves were collected from up to 40 species keeping track of the node, counting from the growing tip of the vine, they were collected from. Species have been clustered into Morphotypes A-G morphologically (not phylogenetically) as described in [23]: Morphotype A–*Passiflora coriacea*, *P*. *misera*; Morphotype B–*P*. *biflora*, *P*. *capsularis*, *P*. *micropetala*, *P*. *organensis*, *P*. *pohlii*, *P*. *rubra*, *P*. *tricuspis*; Morphotype C–*P*. *caerulea*, *P*. *cincinnata*, *P*. *edmundoi*, *P*. *gibertii*, *P*. *hatschbachii*, *P*. *kermesina*, *P*. *mollissima*, *P*. *setacea*, *P*. *suberosa*, *P*. *tenuifila*; Morphotype D–*P*. *amethystina*, *P*. *foetida*, *P*. *gracilis*, *P*. *morifolia*; Morphotype E–*P*. *actinia*, *P*. *miersii*, *P*. *sidifolia*, *P*. *triloba*; Morphotype F–*P*. *alata*, *P*. *edulis*, *P*. *ligularis*, *P*. *nitida*, *P*. *racemosa*, *P*. *villosa*; Morphotype G–*P*. *coccinea*, *P*. *cristalina*, *P*. *galbana*, *P*. *malacophylla*, *P*. *maliformis*, *P*. *miniata*, *P*. *mucronata* (**Fig 1**).

### Comparative morphospace and linear discriminant analysis

To compare grapevine and maracuyá leaves in a common morphospace and perform linear discriminant analysis, a subset of corresponding landmarks was used relative to the Mapper analysis. These points include the base of the petiolar junction, proximal lobe tip, proximal sinus, distal lobe tip, distal lobe sinus, and leaf tip. Both sides of the leaf were sampled in maracuyá. For grapevine, only one side of the leaf was sampled, but for comparison, these points were reflected along the petiolar junction-leaf tip axis to complete the leaf. Leaves were assigned as belonging to ontogenetic or heteroblastic leaf series if the relative node value was less than or equal to or greater than the mean relative node number for a vine, respectively. Leaves were superimposed by generalized Procrustes analysis using the procrustes function from the scipy.spatial module in Python. The sklearn module was used for principal component analysis (PCA) and linear discriminant analysis (LDA). The morphospace was visualized using the PCA inverse_transform function.

### Phylogenetic inference

*Passiflora* spp. sequences from 5 genes (*ITS*, *psbA-trnH*, *trnL*, *trnL-F*, *trnL-T*) were downloaded from GenBank (See S1 Table). We sampled from species that were included in the morphotype dataset. The final dataset comprised of 36 species and 141 sequences. For each gene, sequences were aligned using MUSCLE with the default parameters as implemented in Geneious v.8.0.5 (Biomatters, Auckland, New Zealand). Aligned sequences for each gene were concatenated using Geneious. The final concatenated alignment was used for phylogenetic inference using Bayesian inference. We used GTR substitution for each of the five partitions and ran the MrBayes v.3.2.7 analysis in CIPRES Science Gateway portal [24]. The Monte Carlo Markov chain was run with two runs each of four chains, for 12 million generations with 25% (or 3 million) burn-in and trees sampled every 1000 generations. We considered the convergence of Markovian chains using the log posterior probability and if the effective sample size was ≥300 as analyzed by Tracer v.1.7.2 [25]. We combined the posterior probability of trees using the text editor BBEdit v.14.6.3 (Bare Bones Software; http://www.barebones.com/). Next, we used TreeAnnotator v.1.10.4 (BEAST v.1.10 package; [26]) to generate the maximum clade credibility (MCC) tree using the post-burn-in trees from the combined MrBayes runs (excluding 3 M burn-in trees from each run), with median node heights. The resulting tree was visualized in FigTree v.1.4.4. The selected sequences, concatenated alignment, maximum clade credibility tree and morphotype dataset are in S1–S3 Datasets.

### Stochastic character mapping

Ancestral states estimations of morphotype among sampled *Passiflora* species were estimated and visualized using stochastic character mapping with the make.simmap function [27], under the best fit-model of evolution (ER) determined by the Akaike information criterion in the function fitDiscrete of the R package geiger [28]. One-thousand-character histories were simulated along the maximum clade credibility tree to account for the different character histories and the results were summarized with the plot_simmap function written by Dr Michael May (University of California, Davis & University of California, Berkeley, USA). All analyses were performed in R (R Core Team, 2022). All codes are available at https://github.com/joycechery/PassifloraMorphotype/

### Mapper algorithm

**Fig 2:**
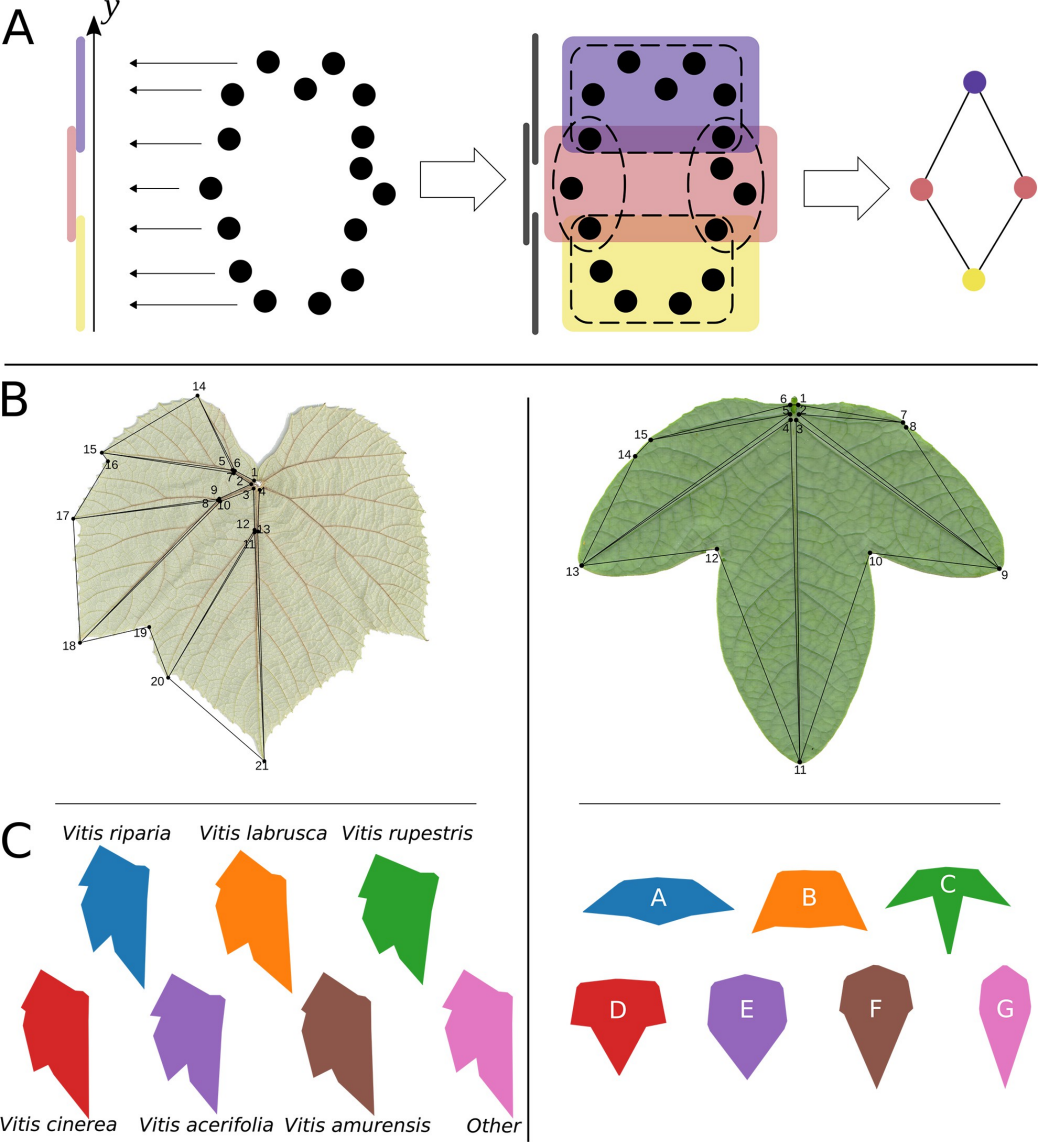
Mapper construction, leaf landmarks, and representative examples from grapevine and maracuyá morphotypes. **(A)** Generalized overview of Mapper graph construction and lens function selection (see **Materials and Methods** for details). Each data point has a distance from every other and is assigned a real number value through a lens function, *y* in this case (left). Points are binned by overlapping cover intervals across the lens function, clustered into nodes, and edges placed between nodes spanning cover intervals with shared data points to create a graph (right). **(B)** Landmarks used for grapevine (left) and maracuyá (right). **(C)** Averaged leaf shapes representing grapevine species (left) and maracuyá morphotypes (right). Note: each species (grapevine) or morphotype (maracuyá) was assigned an arbitrary color which will be used consistently throughout the manuscript.

Leaves in the dataset are represented by 15 or 21 *(x*,*y)*-coordinate pairs for grapevine and maracuyá, respectively, with each pair denoting the location of a landmark (**Fig 2B**). This collection of coordinates can be represented as a 30-dimensional vector, one vector for each leaf. Because direct visualization of such high-dimensional data is impossible, we use the Mapper algorithm to simplify the shape of the data into a one-dimensional graph [17]. For instance, Mapper was recently used to uncover patterns in gene expression in flowering plants which PCA failed to distinguish [29]. The Mapper algorithm works by creating groups of nearby points, then using these groups as a basis for the graph structure. Let X be a point cloud, and consider a *lens function* f: X → ℝ. While the Mapper algorithm is defined for more general maps, in this work, we only consider maps f: X → ℝ. The Mapper algorithm consists of the following steps:

1. Construct an open cover {U_α_} of f(X). In practice, each U_α_ is an interval in ℝ, with endpoints determined by the user-specified number of intervals and their overlap.
2. Cluster the points within each open f^-1^(U_α_).
3. The Mapper graph is the one-skeleton of the nerve of the clustering from the previous step; each cluster corresponds to a vertex in the Mapper graph, with two vertices being connected if there is a point in each of their respective clusters.

The mathematical description above can be described qualitatively, as follows. We think of our data as a point cloud, where the coordinates of each point, or leaf, are determined by the 30-dimensional vector containing the coordinates of each landmark. To use the Mapper algorithm, we must first have a notion of “distance” from any leaf to any other leaf. We choose to use the correlation distance, defined as 1-r, where r is the Pearson correlation coefficient, because in this semi-metric, leaf vectors with high correlation between their landmarks will have a distance near zero (**Fig 2A**).

At a high level, the first step in the Mapper algorithm is to group points with similar points. There are many ways to do this, but the implementation of the Mapper algorithm we use follows a specific protocol: first, we perform a dimensionality reduction step by mapping each leaf to a value determined by a lens function. This lens function is user-defined and is determined by which aspect of the data is the topic of study. In our work, we make use of two lens functions: the first PCA component, and heteroblasty.

Now that the data has been mapped to one dimension, we cover these projected data points with a set of overlapping intervals. The open cover used in the Mapper algorithm is arbitrary and affects the structure of the resulting graph. Heuristically, coarser covers lead to graphs with fewer vertices and finer covers result in graphs with more vertices. Similarly, higher amounts of overlap between cover intervals generally result in graphs with more edges between vertices. In this work, we hand-tune the open cover to obtain a graph that is sufficiently detailed while still being human-readable. Once the intervals in the open cover have been defined, we use these intervals to group points in the original data set: two points are in the same group if their projected images are in the same interval.

The next step is to build the Mapper graph from these groupings. Inside of each group, we perform a clustering algorithm. We use DBSCAN [30] since it does not require the user to select the number of clusters *a priori* and is robust to outliers. We construct the Mapper graph as follows: each cluster becomes a vertex in the Mapper graph. Vertices are connected if both vertices contain a common data point; this is possible because one data point may be in multiple intervals. The resulting graph is a one-dimensional Mapper graph.

## Results

### Mapper resolves relationships within grapevine and maracuyá better than PCA

**Fig 3:**
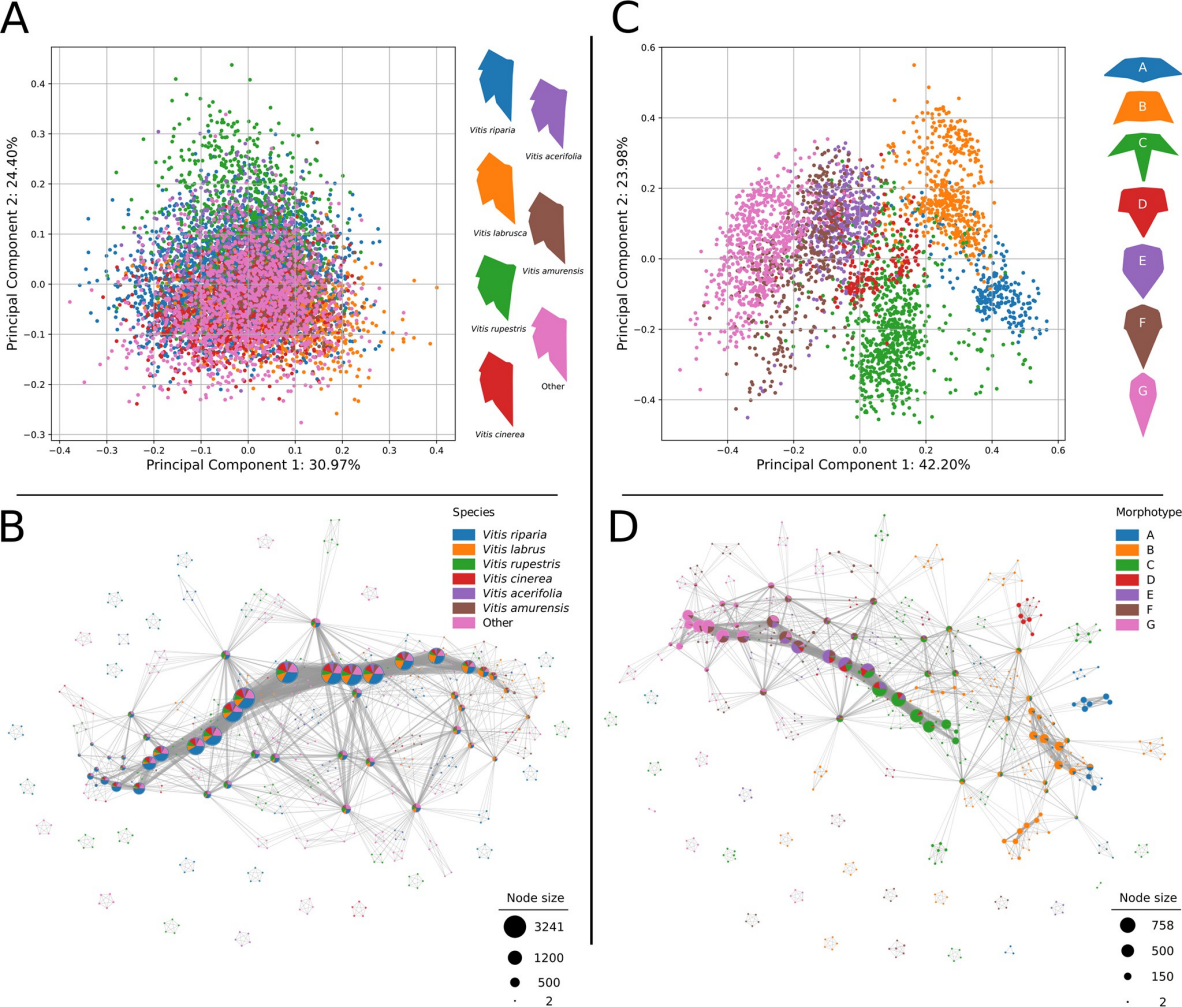
Principal component analyses (PCA) and PC1-derived Mapper graphs of grapevine and maracuyá morphotypes. **(A)** PCA fails to clearly distinguish grapevine morphotypes. **(B)** A Mapper graph using PC1 as a lens retains the underlying structure of the PCA plot and better resolves membership within each group. **(C)** PCA resolves maracuyá morphotypes better than grapevine but shows substantial overlap near the center of the plot. **(D)** A Mapper graph using PC1 as a lens retains the underlying structure of the PCA plot and better resolves membership within each group.

Leaves are high-dimensional shapes derived from the integration of developmental, environmental, and evolutionary forces (**Fig 1**). To make sense of high-dimensional datasets, traditional data analysis often relies on Principal Component Analysis (PCA). We therefore revisited morphological datasets of grapevine and maracuyá to see whether dimension reduction via PCA could provide novel insights into the relationships between these complex and variable leaf shapes (**Figs 1, 3A, and 3B**). PCA plots of maracuyá leaves partially separated the seven morphotypes but still retained significant overlap near the center of the graph (**Fig 3B**). Similar analyses using grapevine leaves were even less successful at distinguishing individual species, with all points essentially clustering into one large group (**Fig 3A**). Thus, while PCA plots provide some separation between groups, they are highly variable and have considerable overlap, obscuring relationships between development, evolutionary, or environmental factors.

As an alternate approach, we turned to the Mapper algorithm, a method that has successfully identified novel relationships within other high-dimensional datasets [19,29,31]. A key advantage of Mapper is its ability to interrogate datasets using several different lenses, revealing structure and relationships from specific, mathematically-defined perspectives that are hidden from traditional approaches (see **Materials and Methods**). First, we tested whether Mapper could recapitulate the relationships between grapevine and maracuyá species identified by PCA. We did this by using Mapper to visualize the structure of the data from the perspective of PC1. By representing vertices in the Mapper graph with pie charts denoting their composition, we were able to better resolve group membership within each vertex for both maracuyá and grapevine (**Fig 3B and 3D**). Further, because PC1 was chosen as the lens, the structure of each Mapper graph retains the underlying structure of its respective PCA plot. For instance, the branches of the maracuyá Mapper correspond to the clusters of its PCA both in relative position and in membership (**Fig 3D**). By contrast, vertices of the grapevine Mapper do not show clear differences in membership, consistent with the single clustered nature of its PCA (**Fig 3B**). Thus, Mapper can recapitulate and improve on results from PCA, and is positioned to identify new relationships that this traditional approach cannot.

### Mapper graphs reveal conserved developmental programs in grapevine and maracuyá

**Fig 4:**
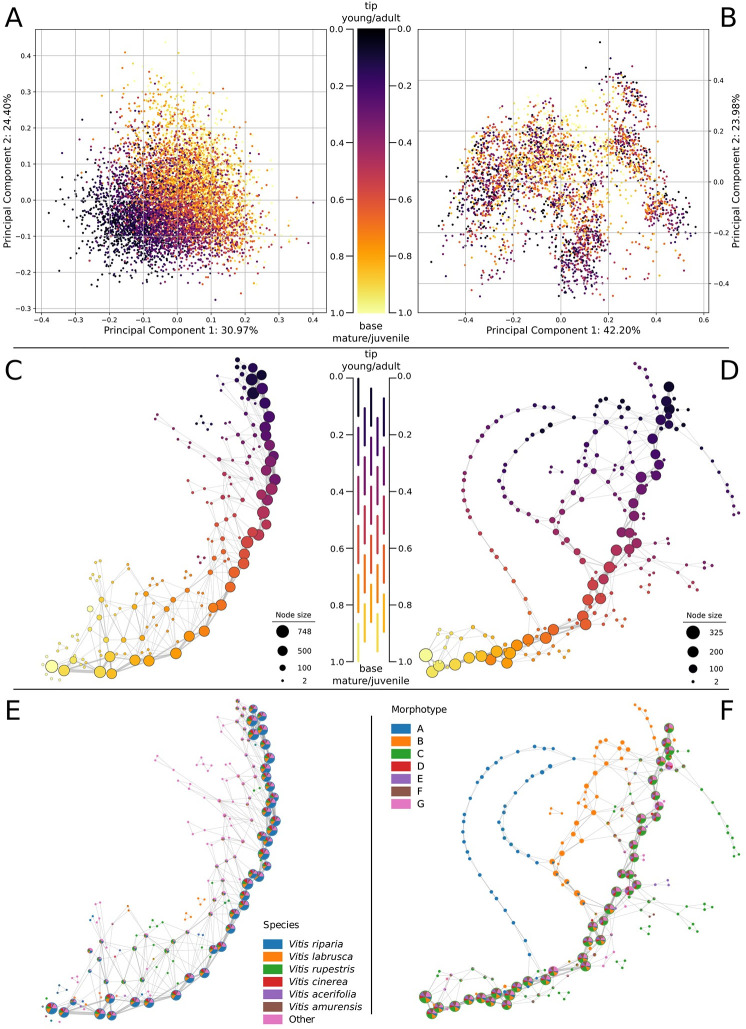
Grapevine and maracuyá species share a core conserved heteroblasty program. **(A)** Recoloring of the grapevine PCA by heteroblasty values suggests a relationship between heteroblasty and both PC1 and PC2. **(B)**. Recoloring the PCA plot for maracuyá yielded no obvious relationships. **(C,E)** A Mapper graph using heteroblasty as a lens reveals a strong central spine in grapevine shared by all morphotypes. **(D,F)** A Mapper graph using heteroblasty as a lens reveals a strong central spine in maracuyá shared by most morphotypes. Two notable exceptions are morphotypes A and B which diverge near the middle of the leaf series before rejoining the central spine.

The maracuyá and grapevine datasets contain several replicates of leaves collected from every node along growing vines. This presents the opportunity to explore developmental questions such as heteroblasty and ontogeny. Further, as the datasets contain several species per genus, these questions can also be examined in an evolutionary context. To begin to address the interplay between evolution, development, and leaf shape, we assigned a relative heteroblasty value to each leaf based on its position, ranging from zero (tip of vine) to one (base of vine). Recoloring the PCA plot for grapevine using these values revealed young, adult leaves cluster near the bottom left of the plot while mature, juvenile leaves cluster towards the top right (**Fig 4A, left**). This suggests a relationship between heteroblasty and both PC1 and PC2, however this relationship is obscured by the many overlapping points on the plot. Recoloring the PCA plot for maracuyá revealed a more complicated distribution, with juvenile and adult leaves displaying a significant amount of overlap and no obvious pattern (**Fig 4, right**). Heteroblastic trends within and between species are thus not clearly delineated using traditional analyses like PCA.

**Fig 5:**
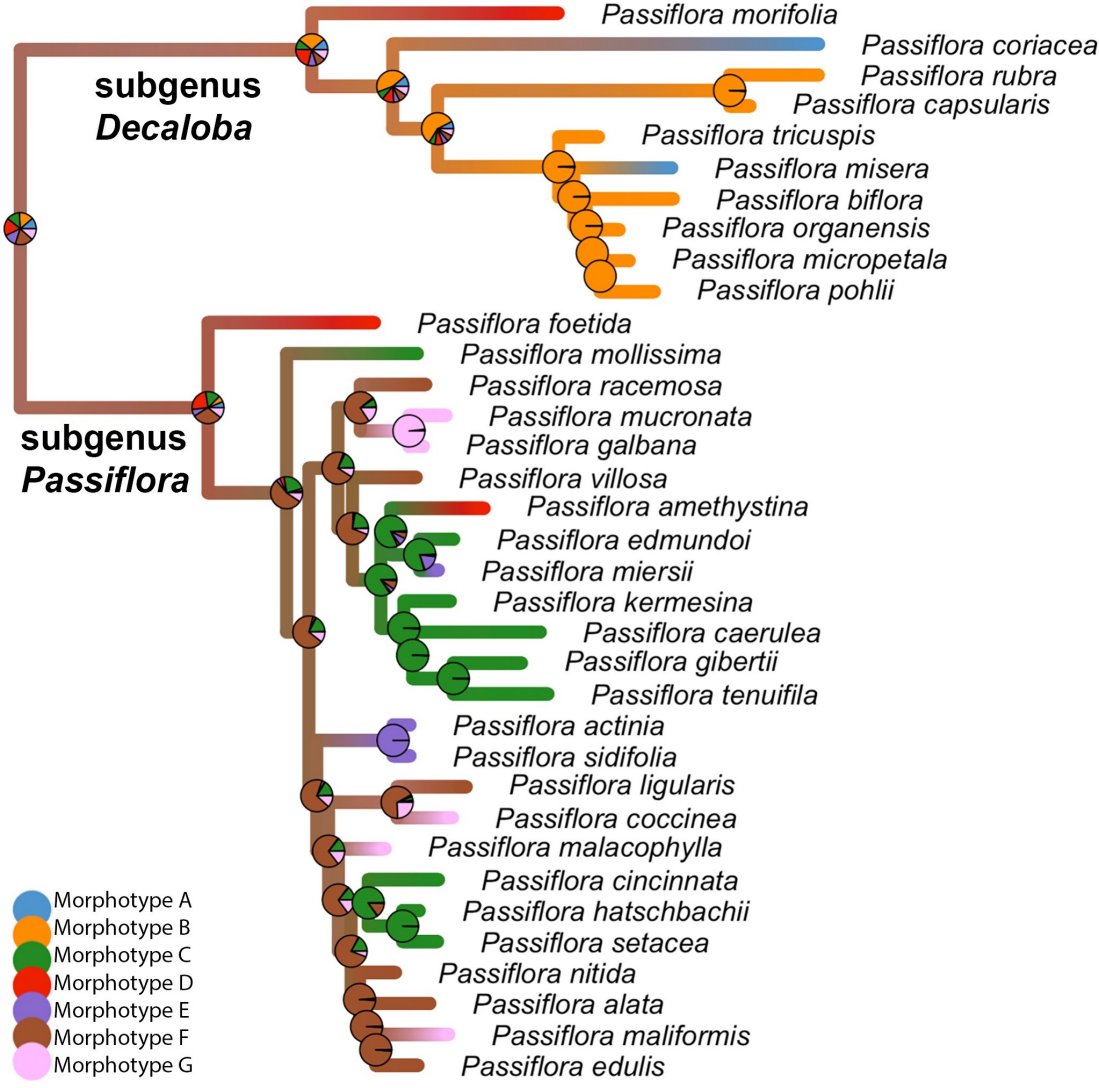
Stochastic character mapping of leaf morphotype evolution along the branches of the *Passiflora* maximum clade credibility tree. Morphotypes A and B (orange and blue) are exclusively in subgenus *Decaloba*. All other species are in subgenus *Passiflora*. Note the repeated independent evolution of morphotypes C through G in subgenus *Passiflora*.

To better identify these hidden relationships, we constructed Mapper graphs for maracuyá and grapevine using heteroblasty as a lens (**Fig 4C and 4D**). Vertices within the resulting Mapper graphs were colored by the average heteroblasty value of each leaf in the vertex. One prediction of the recolored PCA plots is that grapevine should have limited branching, given its relatively clear continuum of heteroblasty values along PC1 and PC2 (**Fig 4A**). Supporting this, the Mapper graph for grapevine consists of a strong central spine, with clear transitions between juvenile and adult leaves, and limited branching (**Fig 4C and 4E**). A second prediction is that maracuyá should have a much more complex structure. Indeed, given their strikingly different leaf morphologies (**Fig 1**), each morphotype could conceivably have its own heteroblastic trajectory (**Fig 4B**). Surprisingly, the Mapper graph for maracuyá also has a strong central spine (**Fig 4D**). As the Mapper algorithm detects shape changes between concurrent nodes, this suggests that most maracuyá species share a core, deeply conserved heteroblasty program despite the markedly different appearances of their leaves (**Fig 1)**. Two interesting exceptions to this are morphotypes A and B which branch off from the central spine at two points before rejoining near the tip (**Fig 4D and 4F**). One potential explanation is the evolution of distinct heteroblasty program(s) in these two morphotypes. To generate additional support for this idea, we performed an ancestral state reconstruction of leaf morphotypes across maracuyá which yielded two key findings **(Fig 5**). First, species in morphotypes highly represented in the central spine (morphotypes C through G) fall into one clade, subgenus *Passiflora*, where these morphotypes appear to have evolved independently numerous times. Mapper can thus detect conservation even in rapidly evolving species with dramatically different leaf morphologies. Second, all species with morphotypes A and B are found in subgenus *Decaloba* **(Fig 5)** which is predicted to have diverged from subgenus *Passiflora* between either ~38.3 [32] or ~40 million years ago [33]. Therefore, this vast evolutionary distance might underlie the emergence of distinct heteroblasty program(s) in morphotypes A and B. Finally, as both morphotypes eventually rejoin the main spine, the beginning and ending of these program(s) would be shared by the entire maracuyá family.

**Fig 6:**
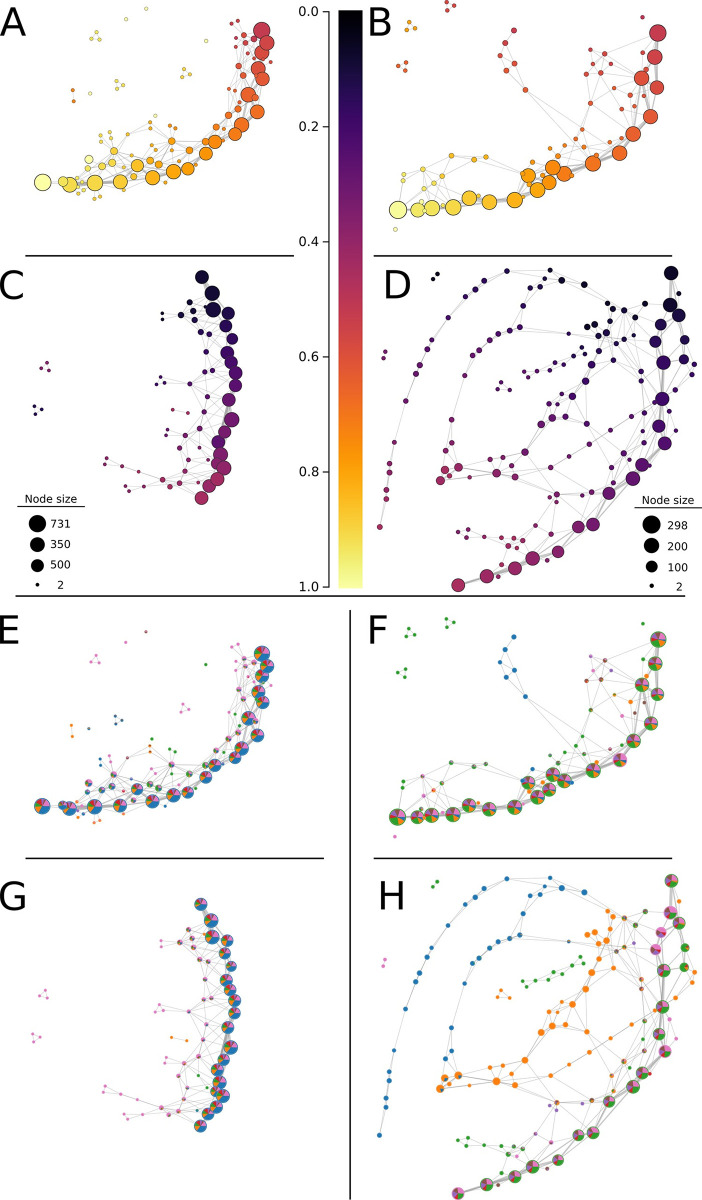
Mapper graphs constructed using partial datasets detect the same signatures as those found using full datasets. **(A,C,E,G)** Mapper graphs generated from partial datasets corresponding to the shoot base (**A,E**) and growing tip (**C,G**) of grapevine closely resemble analogous regions of Mapper graphs generated from the full dataset (**Fig 4C and 4E**). **(B,D,F,H)**. Mapper graphs generated from partial datasets corresponding to the shoot base (**A,E**) and growing tip (**C,G**) of maracuyá closely resemble analogous regions of Mapper graphs generated from the full dataset (**Fig 4C and 4E**).

Topological data analysis using heteroblasty as a lens suggests this developmental process is conserved between species of grapevine or maracuyá (**Fig 4**). One potential caveat concerns measurements taken from the growing tip of vines. As leaves in this region are not fully mature, node-to-node shape changes are influenced by both ontogeny and heteroblasty. Thus, the observed topological signature could be driven by one, or both, of these developmental pathways. To address this, we divided each dataset into shoot base (nodes 0 to 0.5) and growing tip (nodes 0.5 to 1). The former is comprised of mature leaves whose ontogenetic programs have finished, while the latter contains leaves that are still undergoing dramatic allometric shape changes. This allows us to separately interrogate changes conditioned by heteroblasty (i.e. leaves at the base) versus those conditioned largely by ontogeny (i.e. leaves at the growing tip). We note that this approach cannot fully disentangle these developmental programs at the growing tip as final leaf shape may still be partially informed by heteroblasty. Nevertheless, enriching for ontogenetic contributions at the growing tip should help discriminate between heteroblastic versus ontogenetic effects on shape along the vine. We first tested that these partial datasets capture the conserved signature(s) by generating Mapper graphs using heteroblasty as a lens (**Fig 6**). Importantly, these new Mapper graphs closely resemble analogous regions of the Mapper graphs generated using the full dataset (e.g. **Fig 4C** vs **Fig 6A and 6C**; **Fig 4D** vs **Fig 6B and 6D**). This is most striking for maracuyá whose Mapper graphs retain the early branching of morphotype A (**Fig 6F**), the separation of morphotypes A and B from the main spine (**Fig 6F and 6H**), and their eventual rejoining near the tip (**Fig 6H**). These partial datasets suggest there are distinct developmental signatures at the shoot base and growing tip.

**Fig 7:**
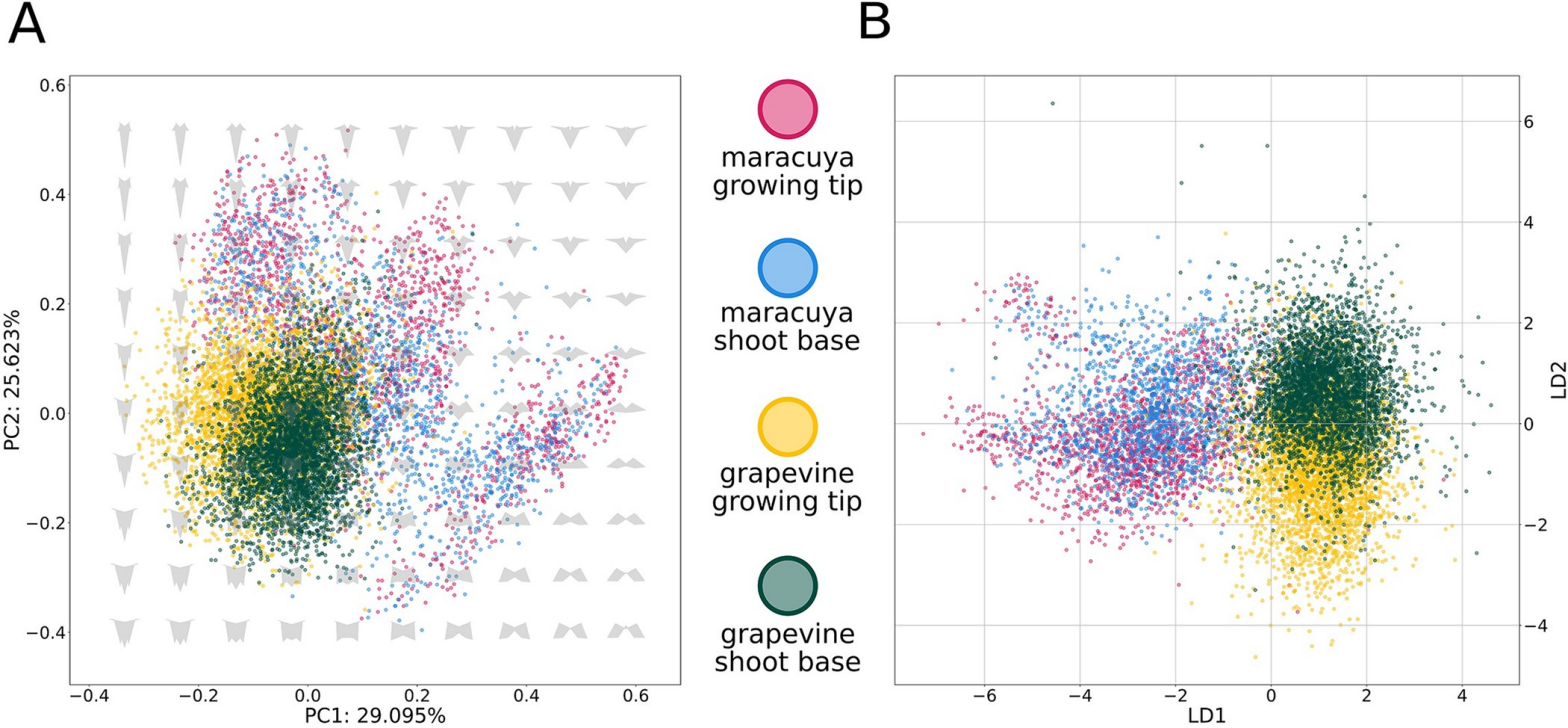
There are distinct signatures of heteroblasty and ontogeny which may be conserved across genera. **(A)** PCA using shoot base and growing tip partial datasets of grapevine (yellow and green) and maracuyá (red and blue). Data points belonging to grapevine and maracuyá do not cleanly separate. Further, separation of shoot base from growing tip along PC1 is evident in grapevine but not in maracuyá. **(B)** LDA using shoot base and growing tip partial datasets of grapevine (yellow and green) and maracuyá (red and blue). Grapevine and maracuyá data points cleanly separate along LD1. Shoot base and growing separate along LD2.

We hypothesized that our topological data analysis was detecting a heteroblastic signature at the shoot base and a predominantly ontogenetic signature at the growing tip. If so, shoot base regions should be more similar to each other than growing tip regions, regardless of species. To test this, we extracted a subset of landmarks shared by both grapevine and maracuyá (see **Methods**). This allowed us to directly compare their shoot bases and growing tips using clustering and dimension reduction analyses. First, in a common PCA containing both grapevine and maracuyá leaves, grapevine leaves from the shoot base and growing tip occupy distinct regions of the morphospace (**Fig 7A**). This supports the notion that the shoot base and growing tip possess distinct developmental signatures–one due to heteroblasty and the other containing ontogenetic contributions–and that these signatures can be easily visualized in these species. By contrast, PCA plots for maracuyá had no clear pattern, showing extensive overlap of the shoot base and growing tip throughout the morphospace (**Fig 7A**). We therefore turned to linear discriminant analysis (LDA), an alternate approach commonly used for classification. We modeled four categorical groups of leaves as a function of their landmark information. The four groups were: grapevine shoot base, grapevine growing tip, maracuyá shoot base, and maracuyá growing tip. Application of LDA revealed a striking separation of species, as well as heteroblastic versus ontogenetic contributions (**Fig 7B**). For instance, LDA plots show a clean separation of grapevine and maracuyá, regardless of developmental stage, along LD1 (**Fig 7B**). LD2, on the other hand, separates shoot base from growing tip, regardless of species (**Fig 7B**). Taken together, our analyses combining topological data analysis, PCA, and LDA suggest ontogenetic contributions to leaf shape can be partially separated from heteroblasty, and that these two developmental processes might be conserved between grapevine and maracuyá, despite their evolutionary distance.

**Fig 8:**
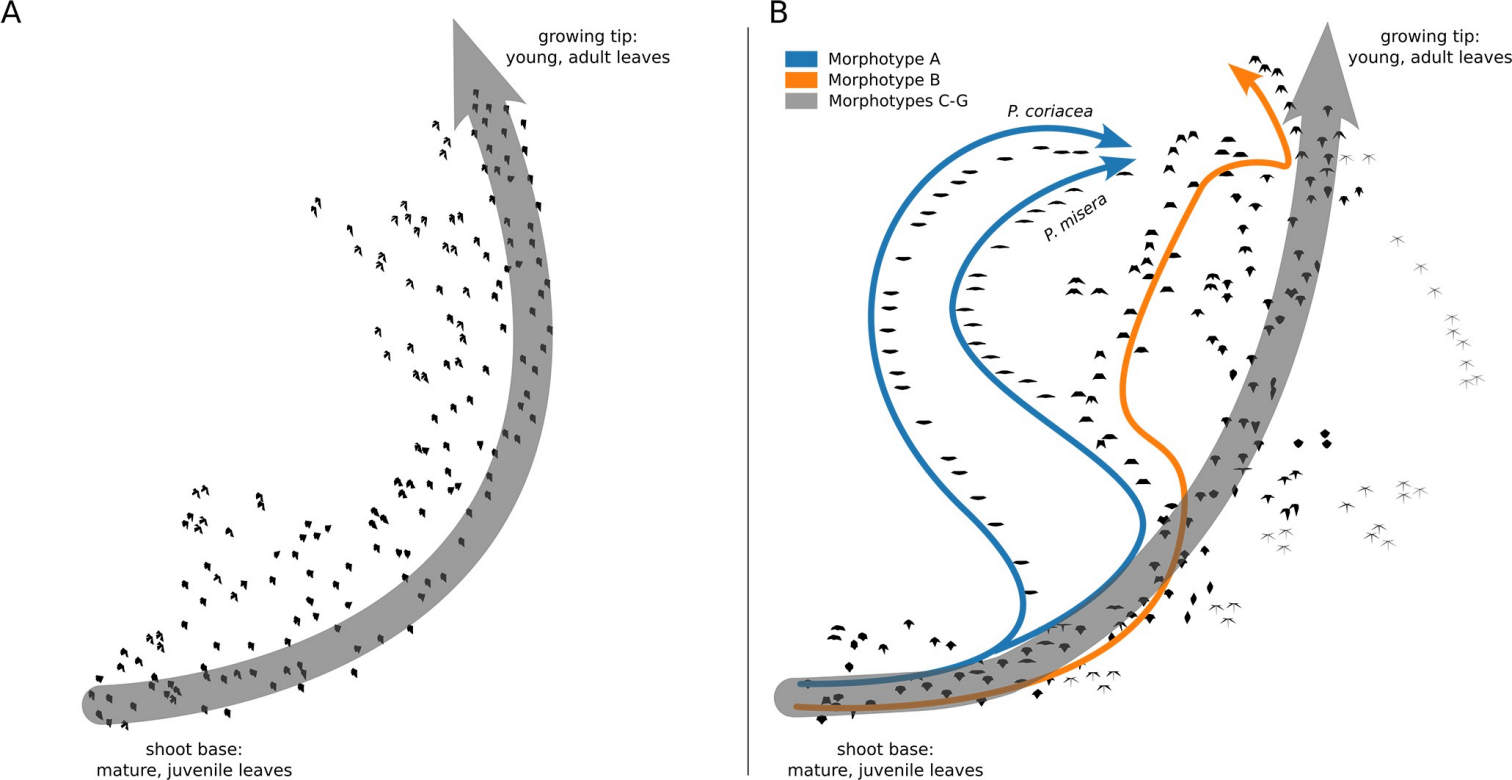
Subway map of grapevine and maracuyá Mapper graphs. **(A)** Primary structure of the grapevine Mapper graph with vertices represented by the average leaf shape within a given vertex. **(B)** Primary structure of the maracuyá Mapper graph with vertices represented by the average leaf shape within a given vertex. No single morphotype dominates each vertex demonstrating that Mapper is clustering species based on an underlying data structure and not by shape similarities. Morphotypes A and B, which display unique heteroblastic trajectories, are labeled in blue and orange, respectively.

The power of Mapper is its ability to visualize hidden structure within high-dimensional datasets which is then presented as an abstract graph composed of edges and vertices. However, to fully understand how shape changes across a given lens (in this case heteroblasty), it is helpful to relate this graphical representation back to the real shapes that drove its construction. To do this, we first extracted the primary structure of the Mapper graphs by highlighting their central spines and branching structures (**Fig 8**). Vertices were then replaced by leaf outlines which represent the average shape of all leaves within a given vertex. The resulting morphospaces provide a tangible illustration of how leaf shape changes along grapevine and maracuyá shoots. For instance, leaf outlines along the central spine of the grapevine Mapper show little deviation, as expected (**Fig 8A**). By contrast, the representative outlines along the central spine of maracuyá are highly variable indicating no single morphotype dominates the morphospace (**Fig 8B**). Thus, despite members of the maracuyá group displaying striking differences in leaf shapes (**Fig 1**), the way their leaves change from node to node is deeply conserved. Exceptions to this–morphotypes A and B–are also visible as branches emerging from different points along the main spine. Whether their evolutionarily distinct nature plays into this branching is an open question.

## Discussion

### Similar developmental trajectories underlie most species of grapevine and maracuyá

Leaves are the product of development, environmental, and evolutionary forces, and their shapes vary dramatically both within plants of a given species and between species of a given family. One example of these forces is a process termed heteroblasty, in which leaves of different shapes are generated from a single plant in an age-dependent manner [4]. In our study, the leaves of the maracuyá family provide a particularly striking illustration of this, though subtle changes can be seen in grapevine, as well (**Fig 1**). Modulation of a core heteroblasty program is thus a logical potential explanation for the species-to-species divergence of leaf morphologies seen between these two families [34,35]. Surprisingly, a Mapper algorithm using a heteroblastic lens revealed that most species, including those in the maracuyá family, have a nearly identical trajectory despite their highly divergent leaf shapes (**Fig 4**). Topological data analysis thus allows the contribution of a given process–in this case heteroblasty–to be isolated from the other forces guiding leaf morphogenesis.

As a caveat to the above, leaves at the base of vines are no longer growing, and it is safe to assume that node-to-node shape changes are dominated by heteroblastic effects. However, leaves at the tip of vines are still undergoing ontogenetic allometric expansion, i.e. changing their shape in response to an underlying ontogenetic program. Node-to-node shape changes in this region could therefore be influenced by both ontogenetic and heteroblastic pathways. Importantly, by separating shoot base from growing tip regions, and coupling topological data analysis to PCA and LDA, we were able to detect signatures from both developmental pathways. Specifically, heteroblasty dominates the base while leaves at the tip have a separate signature carrying at least some contribution from ontogeny. Importantly, LDA in particular suggests the dramatic species-to-species differences in leaf shape are orthogonal to both of these developmental pathways, which may be conserved within and between species of grapevine and maracuyá.

### Modulating leaf shape by coupling slowly and quickly evolving processes

If not heteroblasty or ontogeny, then what other forces or factors might underlie the species-to-species differences in leaf shape seen in these datasets? Multiple molecular pathways intersect to control leaf shape, and there is no shortage of candidates (reviewed in [36]). For instance, leaf complexity is regulated by several transcription factors that in some cases operate independently of heteroblasty. The homeobox gene LATE MERISTEM IDENTITY1 (LMI1) / REDUCED COMPLEXITY (RCO), for example, drives evolutionary differences in leaf shape between species within Brassicaceae [37,38] and between varieties of cotton [39]. Similarly, mutations in KNOX genes can confer evolutionarily labile effects on leaf complexity between closely related species of tomato [40,41]. Regardless of the specific factor, a plausible explanation for species-specific differences in maracuyá might be evolutionarily labile effects of genes regulating leaf morphology, similar to those described above, layered on more slowly evolving developmental pathways such as auxin signaling [42], adaxial-abaxial patterning [43], proximal-distal patterning [44], and miR156-miR172-mediated heteroblasty [45], the effects of which are conserved in maracuyá [46]. Gene regulatory networks distinct from conserved pathways regulating developmental transitions across the flowering plants would be expected to confer different effects on leaf shape and might explain the independence of these two processes we observe at a morphological level.

### A ‘reverse hourglass’ in select maracuyá morphotypes

Two interesting exceptions emerged from our analyses of maracuyá leaf shape. Whereas all maracuyá species cluster at the growing tip and base of their shoots, morphotypes A and B in subgenus *Decaloba* diverge in the middle of the leaf series (**Figs 4 and 5**). One useful metaphor to describe this relates to the hourglass model of gene expression which posits that gene expression programs are more conserved near the middle of embryogenesis than the beginning and end [47]. In the case of subgenus *Decaloba*, our data reveal a “reverse hourglass” effect, with similarity highest near the beginning and end of the leaf series, and lowest near the middle. This suggests that these species deploy the standard maracuyá heteroblastic and/or ontogenetic programs at their tip and base but evolved unique program(s) near the middle of their leaf series. Alternatively, the core developmental programs may be conserved throughout, but other independent effects exert a particularly strong influence in this region in these morphotypes. Genetic and molecular assays could be used to distinguish between these scenarios. Nevertheless, these findings illustrate the ability of topological data analysis to highlight biologically relevant relationships.

### Perspectives

Questions in biology are increasingly driven by large datasets comprised of ecological, morphological, and molecular measurements. These datasets contain enormous amounts of information, far more than many biologists appreciate. For instance, even a single leaf on a growing plant is defined by multiple identities and contains volumes of information. Morphologically, it has length, width, and depth, and is growing along these axes at different, allometric rates. Evolutionarily, it is a member of a particular species with a defined evolutionary history. Ecologically and physiologically, it is located at specific latitude and longitude coordinates and is experiencing a local microclimate. It has also been exposed to a unique combination of environmental conditions over its lifetime. At a more granular level, it is comprised of hundreds of cells clustering into distinct cell types such as epidermis, vasculature, and mesophyll. Each of these cells has its own unique molecular identity defined by chromosomes with specific combinations of epigenetic marks, a transcriptome, a proteome, and a metabolome. Note that these are merely a subset of ways that one could define a single leaf. The central challenge of the field is to make sense of this enormous amount of information. Topological data analysis offers a simple, flexible, and powerful way to meet this challenge, allowing different underlying data structures arising from mathematically-defined perspectives of the same data to be visualized. Moving forward, phenotypic and molecular data structures from the same samples could be directly compared to each other or modeled after each other, discerning previously confounded mechanisms and ultimately permitting the development of predictive models of complex phenotypes from underlying molecular and genetic signatures. Topological data analysis is thus poised to provide transformative insights into a wide range of biological questions.

## Supporting information

S1 Dataset Passiflora_Sequence-alignment. Input file for MrBayes Bayesian Inference phylogenetic analysis. This file is a concatenated alignment of ITS, psbA-trnH, trnL, trnL-trnF, trnL-trnT. Format is a.nex file for ease of reproducibility.
(NEX)

S2 Dataset Passiflora_morphotypes. Species and their designated morphotypes. Format is a csv file for ease of reproducibility.
(CSV)

S3 Dataset Passiflora_maximum_clade_credibility. Maximum clade credibility (MCC) tree using the post-burn-in trees from the combined MrBayes runs (excluding 3 M burn-in trees from each run), with median node heights. Tree generated using TreeAnnotator v.1.10.4 (BEAST v.1.10 package; Suchard et al., 2018)[26]. Format is a.tree file for ease of reproducibility.
(TREE)

S1 Table Passiflora_Sequence-Accessions. Genbank accessions for the sequences utilized for phylogenetic inference.
(XLSX)
